# Genotype-phenotype correlations in retinitis pigmentosa: structural and vascular insights using OCT and OCTA

**DOI:** 10.1186/s40942-026-00882-7

**Published:** 2026-06-24

**Authors:** Melike Balikoglu-Yilmaz, Aydan Kocar, Aslı Subasioglu, Roya Gasimli, Ferhan Elmali

**Affiliations:** 1 Department of Ophthalmology, Faculty of Medicine, Izmir Katip Celebi University, Izmir, Türkiye; 2 Artvin State Hospital, Artvin, Türkiye; 3 Department of Medical Genetics, Faculty of Medicine, Izmir Katip Celebi University , Izmir, Türkiye; 4 Department of Medical Biology, Faculty of Medicine, Ege University , Izmir, Türkiye; 5 Department of Biostatistics, Faculty of Medicine, Izmir Katip Celebi University, Izmir, Türkiye

**Keywords:** Gene variants, Genotype–phenotype correlation, Optical coherence tomography, Optical coherence tomography angiography, Retinitis pigmentosa

## Abstract

**Background:**

This study aimed to identify disease-related genetic variants in 41 patients with retinitis pigmentosa (RP) and to evaluate their phenotypic effects on the retina and choroid.

**Methods:**

Patients diagnosed with RP underwent targeted next-generation sequencing of RP-related genes and mitochondrial DNA analysis, with genetic counseling. Variants were classified according to ACMG guidelines. Central macular thickness (CMT), retinal nerve fiber layer thickness (RNFLT), subfoveal choroidal thickness (SfCT), foveal avascular zone area (FAZa), ellipsoid zone (EZ) integrity, and vascular density (VD) in the superficial capillary plexus (SCP), deep capillary plexus (DCP), and choriocapillaris (CC) were assessed using OCT and OCTA.

**Results:**

Patients were classified into three groups: Group 1 (USH2A; *n* = 11), Group 2 (other single-gene variants; *n* = 21), and Group 3 (multiple-gene variants; *n* = 9). There were no significant differences in age, sex, consanguinity, symptom onset, disease duration, best-corrected visual acuity, intraocular pressure, RNFLT, or EZ integrity. Pairwise comparisons showed a higher preserved EZ rate in Group 2 (66.7%) than in Group 3 (4.8%, *p* = 0.020). Mean SfCT was lower in Group 3 (147.9 ± 107.7 μm) than in Group 2 (243.9 ± 26.3 μm, *p* = 0.046). SCP and DCP FAZa and VD did not differ significantly; inferior-quadrant CC VD was lower in Group 3 than in Groups 1 and 2 (*p* ≤ 0.01).

**Conclusion:**

RP patients harboring more than one variant showed reduced preserved EZ, SfCT, and inferior-quadrant CC VD compared to patients with a single variant. These findings require further investigation in larger cohorts, together with functional studies, to clarify their clinical and biological significance.

## Introduction

Retinitis pigmentosa (RP) is an inherited retinal dystrophy and the most common subtype of retinal degenerations, with an estimated prevalence of approximately 1 in 4,500 individuals [1]. The disease is characterized by progressive degeneration of the retinal pigment epithelium and rod–cone photoreceptors, typically beginning in the peripheral retina and advancing toward the macula and fovea, ultimately leading to significant visual impairment [2–4].

First described by Van Trigt in 1853 and later pathologically characterized by Donders in 1857, RP exhibits a highly heterogeneous genetic landscape [4, 5]. The condition is predominantly caused by diverse monogenic variants; however, digenic–diallelic and digenic–triallelic modes of inheritance have also been reported [6, 7]. Genes associated with RP are involved in multiple cellular pathways essential for photoreceptor survival and function—particularly in rod photoreceptors—including phototransduction, ciliary transport, outer segment structural integrity, RNA splicing, and the retinoid cycle [6]. Syndromic forms of RP are most commonly inherited in an autosomal recessive manner and involve pathogenic variants in nearly 70 genes [7]. Variants in frequently implicated genes such as *USH2A*, associated with Usher syndrome, as well as in other genes primarily linked to autosomal recessive retinal dystrophies (including *ABCA4* and *EYS*), are more prevalent in populations with consanguineous marriages, which increase homozygosity and facilitate clearer genotype–phenotype correlations, often resulting in earlier disease onset and greater severity. In addition, variants in *RHO*, *RPGR*, and other genes disrupt these core pathways through distinct yet converging mechanisms, ultimately leading to progressive photoreceptor degeneration [8, 9]. The first variant identified as causative for RP was the *P23H* variant in the *RHO* gene, accounting for approximately 12% of RP cases [7].

Genetic analysis in patients with RP is essential for accurate differential diagnosis, genetic counseling, prenatal and preimplantation genetic diagnosis, and prognosis prediction. Moreover, detailed genotype–phenotype correlation studies provide critical insights into disease heterogeneity and progression, which manifests at genetic, allelic, phenotypic, and clinical levels [10]; the same variant may result in distinct clinical outcomes even among members of the same family [6]. In this context, next-generation sequencing (NGS) has become one of the most powerful and widely used approaches for identifying germline variants underlying inherited retinal disorders [11].

Advances in ophthalmic imaging have further contributed to the understanding of RP pathophysiology. Non-invasive techniques such as optical coherence tomography angiography (OCTA) enable the assessment of retinal and choroidal microvascular alterations associated with disease progression [12]. However, the relationship between specific genetic subgroups and quantitative retinal microvascular changes on OCTA remains incompletely understood. By integrating targeted NGS with detailed, sectoral OCTA parameters, the present study aims to provide a comprehensive genotype–phenotype analysis, offering novel insights into gene-specific microvascular patterns and highlighting the potential role of OCTA as a non-invasive biomarker for genotype-driven disease stratification in RP.

## Methods

### Study design

The study adhered to the tenets of the Declaration of Helsinki and was approved by the Ethics Committee of Izmir Katip Celebi University (approval number: 0493, date: 26.10.2023). Written informed consent was obtained from all participants prior to inclusion in the study. This retrospective study reviewed the medical records of 41 patients with a clinical diagnosis of RP who had documented genetic analysis results from the Department of Medical Genetics at Izmir Katip Celebi University.

Inclusion criteria were patients aged 14–83 years with a clinically confirmed diagnosis of RP and available genetic analysis results. For optical coherence tomography (OCT) and OCTA analyses, only the right eye of each participant was included. Exclusion criteria included suspicion of hereditary retinal dystrophies other than RP (e.g., Stargardt disease or Best vitelliform macular dystrophy), severe lens or corneal opacities, a history of refractive surgery, intraocular inflammation, dry eye disease, refractive errors less than − 6.00 diopters or greater than + 3.00 diopters, ophthalmic surgery within the previous three months, systemic diseases, or inability to undergo an adequate fundus examination.

The diagnosis of RP was established by experienced retina specialists based on clinical history and comprehensive ophthalmologic examination findings, including characteristic fundus features such as bone-spicule pigmentation, attenuation of retinal vessels, and waxy optic disc pallor. Ancillary examinations, including OCT, fundus autofluorescence, visual field testing, and full-field electroretinography (ffERG), when available, were reviewed to support the diagnosis.

Patients diagnosed with RP underwent targeted NGS of RP-related genes and mitochondrial DNA (mtDNA) analysis, accompanied by genetic counseling. Identified variants were interpreted according to the American College of Medical Genetics and Genomics (ACMG) guidelines. Based on the genetic analysis results, patients were classified into three groups. Group 1 consisted of patients harboring *USH2A* variant (*n* = 11). Group 2 included patients with a variant detected in a single RP-associated gene other than *USH2A* (*n* = 21). Group 3 comprised patients in whom variants were identified in more than one RP-associated gene within the same individual (*n* = 9).

Although 41 patients with genetically confirmed RP were included in the study, OCT and OCTA analyses could be performed in 32 patients with available high-quality imaging data suitable for quantitative evaluation.

### Data collection tools

Demographic data (age and gender), age at symptom onset (ASO), and history of consanguineous marriage were recorded by reviewing the retrospective medical files of 41 patients with a clinical diagnosis of RP. Best-corrected visual acuity (BCVA) was measured using the Snellen chart and converted to logarithm of the minimum angle of resolution (logMAR). Intraocular pressure (IOP) was measured using a non-contact tonometer (TOMEY FT-1000; Tomey Corporation, Nagoya, Japan). All imaging procedures were performed after pharmacological pupil dilation.

Spectral-Domain OCT data were retrieved from the IMAGEnet 6 archive (Topcon Corporation, Tokyo, Japan). Central macular thickness (CMT), subfoveal choroidal thickness (SfCT), and ellipsoid zone (EZ) measurements were obtained using line scan mode in patients with OCT scans demonstrating an image quality score above 70. EZ integrity was evaluated on horizontal SD-OCT B-scans passing through the fovea and classified into four categories according to the continuity and extent of the EZ band. EZ 1 (intact) was defined as a continuous and clearly identifiable EZ line across the foveal region. EZ 2 (absent) was defined as complete loss of the EZ band in the foveal area. EZ 3 (damaged) referred to partial disruption or irregularity of the EZ band with residual continuity remaining. EZ 4 (residual) was defined as a markedly shortened residual EZ segment confined to the foveal region. In eyes with residual EZ, the horizontal EZ width was manually measured in micrometers (µm). CMT and SfCT were calculated using automated segmentation software, while retinal nerve fiber layer thickness (RNFLT) was assessed using peripapillary circular scan protocols.

Optical Coherence Tomography Angiography: Imaging was performed using 6.0 × 6.0 mm macular scan protocols in IMAGEnet 6 (Topcon Corporation, Tokyo, Japan). Vascular density (VD) values were obtained from the nasal, temporal, superior, and inferior quadrants of the superficial capillary plexus (SCP), deep capillary plexus (DCP), and choriocapillaris (CC). The foveal avascular zone area (FAZa) was manually measured in square micrometers (µm²) in both the SCP and DCP. Images with poor quality due to blinking or motion artifacts, in which vascular borders could not be clearly distinguished, were excluded from analysis.

Automatic layer segmentation was used to separate the SCP, DCP, and CC. VD was calculated as the ratio of perfused vessel area to the total scan area (%). FAZa was determined through non-flow area detection, and quantitative assessments were conducted using the density mapping tools available within the software.

### Genetic sequencing and bioinformatic analysis

Clinical targeted NGS was performed using a retinal dystrophy gene panel encompassing 108 genes associated with inherited retinal diseases. This panel included genes representing major functional pathways implicated in RP, such as *ABCA4*, *USH2A*, *RPGR*, and *RHO*, among others involved in shared biological processes essential for retinal homeostasis. The analyzed genes were evaluated according to their known cellular functions and pathogenic roles associated with RP, as summarized in Table 1.

**Table 1:** Functional classification of genes included in the clinical retinal dystrophy panel, illustrating the major molecular pathways and disease mechanisms implicated in retinitis pigmentosa

Functional category	Genes	RP-related mechanism
Phototransduction	RHO (OMIM:180380), PDE6A (OMIM:180071), PDE6B (OMIM:180072), PDE6G (OMIM:180073), CNGA1 (OMIM:123825), CNGB1 (OMIM:600724), CNGB3 (OMIM:605080), GUCY2D (OMIM:600179), SAG (OMIM:181031)	Defective phototransduction cascade and signal termination
Visual cycle / Retinoid metabolism	ABCA4 (OMIM:601691), RPE65 (OMIM:180069), LRAT (OMIM:604863), RDH12 (OMIM:608830), RDH5 (OMIM:601617), RGR (OMIM:600342), RLBP1 (OMIM:180090), RBP3 (OMIM:180290), RBP4 (OMIM:180250)	Impaired retinoid recycling leading to photoreceptor toxicity
Ciliary structure & transport	RPGR (OMIM:312610), RPGRIP1 (OMIM:605446), CEP290 (OMIM:610142), LCA5 (OMIM:611408), IFT140 (OMIM:614620), WDR19 (OMIM:608151), ARL3 (OMIM:604695), ARL6 (OMIM:608845), BBS1 (OMIM:209901), BBS2 (OMIM:606151), TTC8 (OMIM:608132), OFD1 (OMIM:300170), SCLT1 (OMIM:611399), MAK (OMIM:603317), TUB (OMIM:602529)	Disrupted protein trafficking through the connecting cilium
Outer segment structure	PRPH2 (OMIM:179605), ROM1 (OMIM:180721), PRCD (OMIM:610598), PROM1 (OMIM:604365), CDHR1 (OMIM:609502), EYS (OMIM:612424), RP1 (OMIM:603937), RP1L1 (OMIM:608581), SPATA7 (OMIM:609868)	Instability of photoreceptor outer segment discs
RNA splicing	PRPF3 (OMIM:607301), PRPF6 (OMIM:612926), PRPF8 (OMIM:607300), PRPF31 (OMIM:606419), SNRNP200 (OMIM:601664)	Retina-specific vulnerability to spliceosomal dysfunction
Transcriptional regulation	CRX (OMIM:602225), NRL (OMIM:162080), NR2E3 (OMIM:604485), ZNF513 (OMIM:613302)	Altered photoreceptor differentiation and gene expression
RPE function & phagocytosis	MERTK (OMIM:604705), MFRP (OMIM:606227), BEST1 (OMIM:607854), TIMP3 (OMIM:188826), C1QTNF5 (OMIM:608899)	Impaired RPE support and outer segment clearance
Metabolism & oxidative stress	IDH3B (OMIM:604526), HK1 (OMIM:142600), NMNAT1 (OMIM:608700), CERKL (OMIM:608381), MVK (OMIM:251170), TTPA (OMIM:600415), RCBTB1 (OMIM:607347)	Energy imbalance and oxidative damage in photoreceptors
Peroxisomal & lysosomal pathways	PEX1 (OMIM:602136), PEX2 (OMIM:170993), PEX7 (OMIM:601757), PHYH (OMIM:602026), GNPTG (OMIM:607838), HGSNAT (OMIM:610453), CLN3 (OMIM:607042)	Accumulation of toxic metabolites in retinal cells
Extracellular matrix & adhesion	USH2A (OMIM:608400), USH1C (OMIM:605242), RS1 (OMIM:300839), SEMA4A (OMIM:603307)	Disrupted retinal architecture and cell–cell interactions
Lipid signaling & transport	ADIPOR1 (OMIM:607945), CYP4V2 (OMIM:608614), PITPNM3 (OMIM:608921), PLA2G5 (OMIM:601199), FLVCR1 (OMIM:609144)	Altered lipid homeostasis affecting photoreceptor survival
Transcription factors & retinal development	PAX6 (OMIM:607108), ZNF408 (OMIM:616454)	Abnormal retinal development and gene regulation contributing to RP phenotypes
Cytoskeleton & scaffold proteins	WHRN (OMIM:607928)	Disrupted stereocilia and photoreceptor structural support
Chromatin remodeling / DNA damage response	MORC2 (OMIM:616661)	Altered chromatin dynamics and neuronal degeneration affecting retinal cells

### Genetic sequencing protocol

Targeted NGS was performed using Twist Biosciences technology with a RP–specific gene panel. Genomic DNA was fragmented, and approximately 36.5 Mb of Consensus Coding Sequences (CCS) covering > 98% of the human coding genome—corresponding to RefSeq and GENCODE v28 regions—were enriched using the Twist Exome 2.0 capture kit. The prepared libraries were sequenced on the MGI DNBSEQ-G400 platform, achieving a minimum read depth of ≥ 20× for > 98% of the targeted bases. Sequencing generated raw data files in FASTQ format for downstream analysis.

### Data processing and variant interpretation

Bioinformatic analysis of FASTQ files was performed using the Franklin by Genoox platform. Variant filtration incorporated databases including HGMD^®^, ClinVar (RRID: SCR_006169), and CentoMD^®^. Variants with a minor allele frequency (MAF) < 1% in the gnomAD (RRID: SCR_014964) database, and those previously reported as pathogenic or likely pathogenic, were prioritized. All potential inheritance patterns were evaluated, and analysis focused on coding exons and the ± 20 bp flanking intronic regions.

Regions with high GC content, complex structural variation, or read depth < 50× were considered as parameters that could reduce the sensitivity of variant detection.

Variant classification was performed according to the ACMG guidelines [13]. Variants classified as Class 1 (pathogenic), Class 2 (likely pathogenic), and Class 3 [variants of uncertain significance] associated with the patients’ clinical phenotypes were reported. Family history and clinical findings were incorporated as supportive evidence during variant interpretation.

Additional mitochondrial whole-genome sequencing and mitochondrial multiplex ligation-dependent probe amplification (MLPA) analyses were performed in patients without initially identified causative variants; however, no additional pathogenic variants were detected. Therefore, only patients with genetically identified RP-associated variants were included in the final statistical analyses.

### Mitochondrial whole-genome sequencing

Genomic DNA was isolated from peripheral blood samples using the QIAcube HT automated system, and DNA concentration was measured with Qubit™ fluorometric quantification. NGS libraries were prepared using the QIAseq kit (QIAGEN) and sequenced on the Illumina MiSeq platform. Bioinformatic processing was conducted using CLC Genomics Workbench, and variant interpretation was performed within the Franklin by Genoox analysis environment. Mitochondrial variants were classified according to the ClinVar, MITOMAP, and MitoPhen databases.

### Mitochondrial multiplex ligation-dependent probe amplification

DNA isolation was performed using the QIAcube HT system, and DNA concentration was determined by Qubit™ measurement. DNA denaturation and probe hybridization were carried out in accordance with standard MLPA procedures. Following ligation and PCR amplification, fragment analysis was performed using an ABI 3500 genetic analyzer, and data interpretation was conducted with Coffalyser software.

### Statistical analysis

Statistical analyses were conducted using SPSS version 30 (SPSS Inc., Chicago, IL, USA). Categorical variables were summarized as frequencies and percentages, while continuous variables were expressed as mean ± standard deviation for normally distributed data and as median (interquartile range, Q1–Q3) for non-normally distributed data. Normality was assessed using the Shapiro-Wilk test, and homogeneity of variances was evaluated with the Levene test. Comparisons between groups were performed using one-way analysis of variance (ANOVA) for normally distributed variables and Kruskal-Wallis tests for variables not meeting normality assumptions. Post hoc multiple comparisons following ANOVA were performed using the Tukey test. For categorical variables, group comparisons were conducted with the Fisher-Freeman-Halton exact test. When significant differences were detected, subgroup analyses were performed using Bonferroni-corrected two-proportion z-tests. Multiple testing corrections was applied where necessary to avoid false positives. Correlations between continuous variables were assessed using Pearson’s or Spearman’s correlation coefficients, as appropriate. A p-value of < 0.05 was considered statistically significant.

## Results

The study included 41 patients with RP, with a mean age of 48.9 ± 16.2 years; 21 were female (51.2%) and 20 were male (48.8%). A history of consanguineous marriage was reported in 21 patients (51.2%). The mean ASO was 31.6 ± 15.9 years.

Next-generation sequencing – based analysis identified causative genetic variants in all patients. Based on the variant profile, patients were classified into three groups: Group 1, patients with *USH2A* variants (*n* = 11); Group 2, patients with other single-gene variants (*n* = 21); and Group 3, patients with multiple-gene variants (*n* = 9). The complete list of identified genes is summarized in Table 2.

**Table 2:** Genetic characteristics of patients with retinitis pigmentosa

Case No.	Gene (Transcript)	Location	Nucleotide (Protein) dbSNP	Zygosity	Variant	ACMG variant classification by Franklin by QIAGEN	Mutation Type
1	USH2A NM_206933.4	Exon 63	c.13,576 C > T p.Arg4526* rs1003869920	Homozygous	Class 1	PVS1, PM3, PM2, PP5	Nonsense/Loss of function
2	USH2A NM_206933.4	Exon 17	c.3502dup p.Thr1168Asnfs*35	Homozygous	Class 1	PVS1, PM2, PP5	Frameshift/Loss of function
3	USH2A NM_206933.4	Exon 17	c.3502dup p.Thr1168Asnfs*35	Homozygous	Class 1	PVS1, PM2, PP5	Frameshift/Loss of function
4	USH2A NM_206933.4	Exon 6	c.908G > A p.Arg303His rs371777049	Homozygous	Class 1	PM3, PM2, PM5, PM1, PP5	Missense
5	USH2A NM_206933.4	Exon 38	c.7174_7181del p.Glu2392Profs*23	Homozygous	Class 2	PVS1, PM2	Frameshift/Loss of function
6	USH2A NM_206933.4	Exon 17	c.3502dup p.Thr1168Asnfs*35	Homozygous	Class 1	PVS1, PM2, PP5	Frameshift/Loss of function
7	USH2A NM_206933.4	Exon 6	c.908G > A p.Arg303His rs371777049	Homozygous	Class 1	PM3, PM2, PM5, PM1, PP5	Missense
8	USH2A NM_206933.4	Exon 6	c.908G > A p.Arg303His rs371777049	Homozygous	Class 1	PM3, PM2, PM5, PM1, PP5	Missense
9	USH2A NM_206933.4	Exon 17	c.3502dup p.Thr1168Asnfs*35	Homozygous	Class 2	PVS1, PM2, PP5	Frameshift/Loss of function
10	USH2A NM_206933.4	Exon 63	c.12580T > C p.Cys4194Arg rs769001387	Homozygous	Class ½ (conflicting pathogenity)	PP5, PM2, PM1, BP4	Missense
11	USH2A NM_206933.4	Exon 44	c.8740 C > T p.Arg2914* rs766590491	Homozygous	Class 1	PVS1, PM3, PM2, PP5	Nonsense/Loss of function
12	PRPF31 NM_015629.4	Exon 3	c.238G > A p.Val80Met	Heterozygous	Class 2	PM2, PP3, PP2	Missense
13	PRPF31 NM_015629.4	Exon 3	c.238G > A p.Val80Met	Heterozygous	Class 2	PM2, PP3, PP2	Missense
14	PRPF31 NM_015629.4	Intron 10	c.1073 + 6G > A rs765404848	Heterozygous	Class 3	PM2, BP4	Missense
15	EYS NM_001142800.2	Exon 26	c.4665_4666del p.Ala1557Asnfs*6 rs1180060216	Homozygous	Class 2	PVS1, PM2, PP5	Frameshift/Loss of function
16	EYS NM_001142800.2	Exon 4	c.490 C > T p.Arg164* rs794727631	Homozygous	Class 1	PVS1, PM3, PM2, PP5	Nonsense/Loss of function
17	EYS NM_001142800.2	Exon 4	c.426_427insCC p.Val143Profs*24	Homozygous	Class 2	PVS1, PM2	Frameshift/Loss of function
18	PDE6A NM_000440.3	Exon 1	c.304 C > A p.Arg102Ser rs141252097	Homozygous	Class 1	PM3, PM2, PM5, PP5	Missense
19	PDE6A NM_000440.3	Exon 1	c.304 C > A p.Arg102Ser rs141252097	Homozygous	Class 1	PM3, PM2, PM5, PP5	Missense
20	IMPG2 NM_016247.4	Exon 2	c.311_312del p.His104Argfs*5 rs1400886227	Homozygous	Class 2	PVS1, PM2	Frameshift/Loss of function
21	IMPG2 NM_016247.4	Exon 2	c.311_312del p.His104Argfs*5 rs1400886227	Homozygous	Class 2	PVS1, PM2	Frameshift/Loss of function
22	MAK NM_001242957.3	Exon 6	c.485 C > T p.Thr162Ile rs774229391	Homozygous	Class 2	PM3, PP3, PM2, PP5	Missense
23	MAK NM_001242957.3	Exon 2	c.79G > A p.Gly27Arg rs754916169	Heterozygous	Class 1	PS1, PM3, PM2, PP1, PP5	Missense
		Exon 6	c.485 C > T p.Thr162Ile rs774229391	Heterozygous	Class 2	PM3, PP3, PM2, PP5	Missense
24	ZNF408 NM_024741.3	Exon 5	c.1163_1174del p.Lys388_Ser392delinsThr	Homozygous	Class 3	PM2, PM4	In-frame indel
25	CA4 NM_000717.5	Exon 4	c.391G > A p.Asp131Asn rs370640952	Heterozygous	Class 3	PM2, BP4	Missense
26	WHRN NM_015404.4	Exon 1	c.325dup p.Tyr109Leufs*70	Homozygous	Class 2	PVS1, PM2	Frameshift/Loss of function
27	ADIPOR1 NM_015999.6	Exon 6	c.725T > C p.Val242Ala	Heterozygous	Class 3	PM2	Missense
28	POMGNT1 NM_017739.4	Exon 6	c.461 C > A p.Pro154His rs886043030	Homozygous	Class 3	PM2, PP3	Missense
		Exon 7	c.550 C > T p.His184Tyr rs746638187	Homozygous	Class 3	PM2	Missense
29	RPE65 NM_000329.3	Exon 10	c.1020 C > G p.Tyr340*	Homozygous	Class 2	PVS1, PM2	Nonsense/Loss of function
30	CRB1 NM_201253.3	Exon 9	c.3541T > A p.Cys1181Ser rs62636291	Homozygous	Class 1	PS4, PM2, PM5, PP3, PM1, PP2, PP5	Missense
31	ABCA4 NM_000350.3	Exon 42	c.5882G > A p.Gly1961Glu rs1800553	Heterozygous	Class 2	PS3, PM2, PM5, PP3, PP2, PP5	Missense
32	NR2E3 NM_014249.4	Exon 6	c.932G > A p.Arg311Gln rs28937873	Heterozygous	Class 1	PS4, PM2, PM5, PM1, PP5	Missense
		Exon 7	c.1049 A > G p.Gln350Arg rs756678889	Heterozygous	Class 2	PS4, PM2, PP5	Missense
33	PRPF6 NM_012469.4	Exon 20	c.2638_2639delinsGC p.Phe880Ala	Heterozygous	Class 3	PM2, PP2	In-frame indel
	RP1 NM_006269.2	Exon 2	c.403G > A p.Ala135Thr rs755833719	Heterozygous	Class 3	PM2, BP4	Missense
34	CNGB1 NM_001297.5	Exon 26	c.2503T > C p.Cys835Arg rs773377435	Homozygous	Class 2	PP3, PM2	Missense
	BEST1 NM_004183.4	Exon 10	c.1409 C > T p.Thr470Met rs776730181	Heterozygous	Class 3	PM2, PP2	Missense
35	PDE6B NM_000283.4	Exon 13	c.1670 A > G p.His557Arg rs536742386	Heterozygous	Class 2	PS4, PM2, PM5, PP3, PM1, PP5	Missense
	MERTK NM_006343.3	Intron 17	c.2350–2 A > C	Heterozygous	Class 2	PVS1, PM3, PM2, PP5	Splice acceptor/ Loss of function
36	ABCA4 NM_000350.3	Exon 42	c.5882G > A p.Gly1961Glu rs1800553	Heterozygous	Class 2	PS3, PM2, PM5, PP3, PP2, PP5	Missense
	USH2A NM_206933.	Intron 41	c.8224–2 A > G	Heterozygous	Class 2	PVS1, PM2	Splice acceptor/ Loss of function
37	CYP4V2 NM_207352.4	Intron 4	c.604 + 4 A > G	Homozygous	Class 3	PP3, PM2	Splice region/ Splice-related
	IMPDH1 NM_000883.4	Exon 1	c.62 C > G p.Pro21Arg	Heterozygous	Class 3	PM2, BP4	Missense
	ABCA4 NM_000350.3	Exon 44	c.6103 C > G p.Leu2035Val	Heterozygous	Class 2	PM2, PM5, PM1, PP3, PP2	Missense
38	RHO NM_000539.3	Exon 3	c.659T > G p.Phe220Cys rs766161322	Heterozygous	Class 2	PM2, PM1, PP3, PP2	Missense
	CRB1 NM_201253.3	Exon 4	c.924dup p.Pro309Thrfs*5	Homozygous	Class 2	PVS1, PM2	Frameshift/Loss of function
39	WDR19, NM_025132.4	Exon 25	c.2740G > A, p.Ala914Thr	Homozygous	Class 2	PP3, PM2, PM5	Missense
	GUCY2D, NM_000180.4	Exon 6	c.1495G > C, p.Gly499Arg, rs75126391	Heterozygous	Class 3	PM2	Missense
40	RDH12 NM_152443.3	Exon 6	c.379G > T p.Gly127* rs104894474	Homozygous	Class 1	PVS1, PM3, PM2, PP5	Nonsense/Loss of function
	EYS NM_001142800.2	Intron 13	c.2137 + 1G > A rs199740930	Heterozygous	Class 1	PM3, PVS1, PM2, PP5	Splice donor/ Loss of function
41	MORC2 NM_001303256.3	Exon 6	c.394 C > T p.Arg132Cys rs1064795559	Heterozygous	Class 1	PS2, PP3, PM2, PM5, PP2, PS3, PP5	Missense
	PAX6 NM_001368894.2	Exon 14	c.1267G > A p.Gly423Arg rs1259731819	Heterozygous	Class 3	PM2, PP3, PP2	Missense
	RHO NM_000539.3	Exon 2	c.434 A > T p.Asn145Ile	Heterozygous	Class 3	PM2, PM1, PP2	Missense

**Fig. 1:**
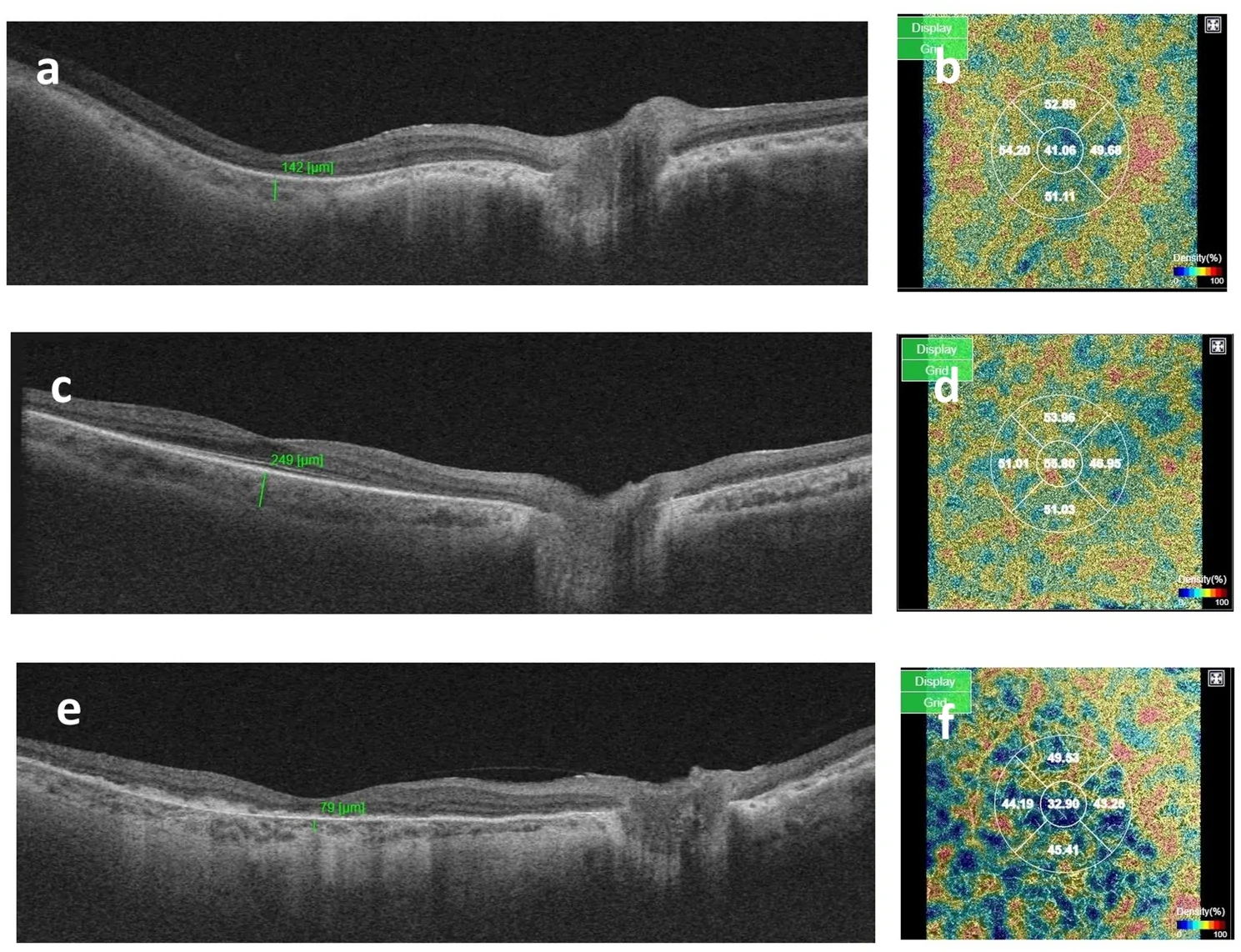
Representative images of subfoveal choroidal thickness (SfCT), ellipsoid zone (EZ), and choriocapillaris (CC) vascular density (VD) in patients with different genetic variants. Subfigures (**a**) and (**b**) show a patient with a USH2A variant; subfigures (**c**) and (**d**) show a patient with another single-gene variant (PRPF31); and subfigures (**e**) and (**f**) show a patient with multiple gene variants. The patient carrying multiple gene variants demonstrates reduced SfCT, disrupted EZ, and decreased CC VD compared to the single-gene variant cases

In Table 3, the only statistically significant difference among groups was observed for EZ integrity. The proportions of “Absent” and “Impaired EZ” did not differ significantly between groups. The residual EZ rate in Group 3 was significantly lower than in Groups 1 and 2, whereas no significant difference was observed between Groups 1 and 2. There was no statistically significant difference in SfCT among the three groups; however, Group 3 exhibited the lowest SfCT values (*p* = 0.261; Table 3; Fig. 1).

**Table 3:** Baseline demographic, clinical, and OCT characteristics of RP Patients according to gene variant groups

Parameter	Group 1 (*n* = 8)	Group 2 (*n* = 19)	Group 3 (*n* = 5)	*P* Value
Age (years)	48.4 ± 13.4	47.7 ± 18.2	53.4 ± 14.1	0.793^†^
Gender, n (Female/Male)	5/3	9/10	3/2	0.792^¥^
Consanguineous Marriage, n (Yes/No)	5/3	12/7	1/4	0.243^¥^
Disease duration (years)	15.5 (8.3–26.3)	10.0 (7.0–16.0)	15.0 (10.0–20.5)	0.314 ^&^
ASO (years)	31.0 (16.5–49.5)	30.0 (18.0–50.0)	30.0 (16.0–45.0)	0.986 ^&^
BCVA (logMAR)	0.5 (0.3–0.5)	0.3 (0.2–0.5)	1.6 (0.0–2.3)	0.450 ^±^
CMT (µm)	193.0 (129.0-221.3)	195.0 (177.0-235.0)	140.0 (88.5–223.0)	0.515 ^±^
SfCT (µm)	215.5 (160.0–277.3)	251.0 (202.0–287.0)	210.0 (60.5–257.0)	0.261 ^±^
RNFLT (µm)	76.0 (55.0–87.0)	68.0 (42.0–88.0)	64.0 (40.0–72.0)	0.537 ^±^
EZ Integrity, n (%)				**0.026** ^¥^
2	2 (25.0)	4 (21.1)	3 (60.0)	
3	0 (0.0)	2 (10.5)	2 (40.0)	
4	6 (75.0)^*a*^	13 (68.4)^*a*^	0 (0.0)^*b*^	

n: number of patients, %: column percentage. Summary statistics for continuous variables are given as mean ± standard deviation for normally distributed data and as median (first quartile–third quartile, Q1–Q3) for non-normally distributed data. Statistical tests: ^(†)^: One-way analysis of variance (ANOVS), ^(&)^: Kruskal-Wallis test, ^(¥)^: Fisher-Freeman-Halton exact test.EZ groups (2/3/4) were classified as Absent / Impaired / Residual, respectively. Residual EZ width was measured only in patients with EZ integrity 4. Comparisons among EZ groups were performed at a significance level of *P* < 0.05. Subgroup analyses for EZ integrity were conducted using the Bonferroni-corrected two-proportion z-test. Superscripts a and b indicate significant between-group differences in residual EZ. The residual rate in Group 3 was significantly lower than in Groups 1 and 2, whereas no significant difference was observed between Groups 1 and 2.Statistically significant p-values are shown in bold.ASO: age at symptom onset, BCVA: best corrected visual acuity, CMT: central macular thickness, SfCT: subfoveal choroidal thickness, RNFLT: retinal nerve fiber layer thickness, EZ: ellipsoid zone

Comparison of OCTA parameters among the three gene variant groups revealed no significant differences in FAZa or VD measurements at the level of the SCP and DCP (all *P* > 0.05, Table 4). In contrast, CC VD showed significant intergroup differences in the superior and inferior sectors. Post-hoc analysis demonstrated significantly lower superior and inferior CC VD in Group 3 compared with both Group 1 and Group 2 (*P* = 0.015 and *P* = 0.002 for superior; *P* = 0.003 and *P* < 0.001 for inferior, respectively), while no significant difference was observed between Groups 1 and 2 (Fig. 1).

**Table 4:** Comparison of OCTA Parameters among patient groups stratified by gene variant groups

Parameter	Group 1 (*n* = 8)	Group 2 (*n* = 19)	Group 3 (*n* = 5)	*P* Value	Post-hoc
SCP FAZa	414.1 (345.2-537.6)	443.6 (371.6-520.3)	613.2 (251.2–929.6)	0.806	-
DCP FAZa	509.3 (425.2-696.1)	562.2 (446.1-754.2)	498.2 (385.8–888.1)	0.888	-
SCP VD Nasal	35.8 ± 3.0	36.7 ± 6.0	36.8 ± 3.3	0.906^†^	-
SCP VD Superior	37.7 ± 3.0	37.3 ± 4.1	36.0 ± 5.1	0.748^†^	-
SCP VD Temporal	37.5 ± 2.8	39.6 ± 5.1	38.6 ± 5.0	0.560^†^	-
SCP VD Inferior	37.4 ± 3.6	35.5 ± 2.8	35.0 ± 1.7	0.234^†^	-
DCP VD Nasal	38.9 ± 4.7	37.1 ± 5.0	38.7 ± 3.3	0.621^†^	-
DCP VD Superior	41.2 ± 4.0	40.5 ± 4.7	40.4 ± 3.8	0.921^†^	-
DCP VD Temporal	38.9 ± 4.0	39.1 ± 5.0	38.8 ± 3.5	0.993^†^	-
DCP VD Inferior	39.2 ± 4.1	38.6 ± 5.0	36.2 ± 2.5	0.489^†^	-
CC VD Nasal	48.8 ± 7.6	51.4 ± 4.2	47.0 ± 7.9	0.267^†^	-
CC VD Superior	51.7 ± 3.9	52.6 ± 2.8	44.6 ± 8.1	**0.002** ^†^	1–2: 0.855 1–3: **0.015** 2–3: **0.002**
CC VD Temporal	52.7 ± 3.3	51.1 ± 3.6	47.9 ± 9.2	0.222^†^	-
CC VD Inferior	51.3 ± 2.1	52.6 ± 3.4	44.2 ± 4.9	**< 0.001** ^†^	1–2: 0.646 1–3: **0.003** 2–3: <**0.001**

n: number of patients, SCP: superficial capillary plexus, DCP: deep capillary plexus, CC: choriocapillaris, FAZa: foveal avascular zone area, VD: vascular density. Summary statistics for numerical variables are presented as mean ± standard deviation for normally distributed variables and as median (first quartile value – third quartile value) for non-normally distributed variables. ^(†)^: One-way analysis of variance; ^(&)^: Kruskal–Wallis analysis. A significance level of *P* < 0.05 was considered statistically significant. Post hoc analyses were performed using the Tukey test.Statistically significant p-values are shown in bold

Spearman correlation analysis was performed to evaluate the relationships between BCVA (logMAR), ASO, disease duration, and OCT parameters within each group. In Group 1, a strong negative correlation was observed between SfCT and ASO (*r* = − 0.750, *p* = 0.008). In Group 2, BCVA (logMAR) demonstrated a positive correlation with ASO (*r* = 0.441, *p* = 0.045), while residual EZ width showed a negative correlation with disease duration (*r* = − 0.597, *p* = 0.024). In Group 3, no statistically significant correlations were identified among the tested variables (*p* > 0.05).

Correlation analysis was performed between BCVA (logMAR) and OCTA parameters within the gene variant groups. In Group 2, a significant negative correlation was observed between BCVA and SCP VD in the temporal quadrant (*r*=-0.54, *p* = 0.02), as well as between BCVA and CC VD in the temporal quadrant (*r* = − 0.54, *p* = 0.02). No significant correlations were detected between DCP VD and other quadrants (nasal, superior, inferior) (*p* > 0.05). In Groups 1 and 3, no significant correlations were observed in any quadrant (*p* > 0.05).

Among the 32 patients in whom variants were identified in only one gene, the variant types were distributed as follows: 16 missense, 10 frameshift, 2 nonsense, 1 splice site, 1 frameshift and nonsense, 1 frameshift and missense, 1 indel, and 1 non-frameshift. When comparing the missense and frameshift subgroups, which included the largest numbers of patients, no significant differences were observed in BCVA (logMAR), OCT parameters (CMT, SfCT, RNFLT, EZ integrity), or OCTA metrics, including FAZa in SCP and DCP, and VD in SCP, DCP, and CC across all quadrants (nasal, superior, temporal, inferior) (*p* > 0.05).

Finally, additional ocular pathologies were evaluated in the RP cohort. Macular edema was present in 5 patients (4 females, 1 male; associated genes: *PRPF31*, *USH2A*, *EYS*, *NR2E3*, *POMGNT1*), and epiretinal membrane (ERM) in 2 patients (2 males; associated genes: *WHRN*, *MAK*.

## Discussion

While several studies have demonstrated structural and vascular changes in RP using OCT and OCTA [14–17], to the best of our knowledge, no studies have specifically compared the effects of different variants on the phenotype. In this context, the present study analyzed the genotypes of RP patients and aimed to contribute to the literature by elucidating differences in their impact on the severity of anatomical and microvascular abnormalities.

In their exhaustive review on RP, Hartong et al. underscored the genetic heterogeneity of the disease, noting that more than 70 genes have been associated with RP [18]. In the present study, the USH2A gene was identified as the most frequently affected, accounting for 26.8% of the observed cases. The study also investigated the role of consanguinity, a genetic factor that has been previously identified. Consanguinity was observed in 51.2% of patients, which may support the hypothesis that autosomal recessive inheritance contributes to RP. Elevated rates of consanguinity have the potential to augment the prevalence of homozygous mutations, a factor that could contribute, at least in part, to the predominance of USH2A variants observed within this cohort. However, no significant associations were observed between consanguinity and BCVA, OCT, or OCTA parameters. These findings suggest that, although consanguinity may increase the risk of disease onset, it does not appear to directly influence phenotypic severity.

USH2A variants represent a significant proportion of autosomal recessive retinitis pigmentosa (arRP) and Usher syndrome type II cases. For instance, c.2299delG has been identified as a causative agent of progressive vision loss accompanied by hearing impairment [19]. Antropoli et al. [20] compared 18 RP patients with *USH2A* variants by classifying them into syndromic (sRP) and nonsyndromic (nsRP) groups. In their study, nsRP patients (*n* = 5) were on average older than sRP patients (*n* = 13), with median ages of 43 years and 33 years, respectively, and the median VMT in all patients was 0.1 logMAR. They reported that the mean SfCT was similar in both groups (182.4 μm in the nsRP group vs. 298.5 μm in the sRP group) and found no significant differences in other quantitative OCT and OCTA parameters. Furthermore, patients diagnosed with Usher syndrome type II demonstrated comparable choroidal parameters (SfCT and choroidal vascularity index [CVI]) and microvascular alterations when compared to nsRP patients, notwithstanding their relatively younger age. A negative correlation between BCVA and DCP VD has also been reported in *USH2A*-related RP patients [20, 21].

Falsini et al. [22] evaluated disease severity in 23 patients with USH2A-related RP using age and electrophysiological parameters. Disease severity was assessed based on BCVA, visual field findings, EZ width, ffERG, and sub-microvolt 30-Hz ERG amplitudes. The authors reported that clinical severity and functional loss increased with age, whereas EZ width and FERG amplitudes progressively decreased during disease progression. These findings support an age-dependent pattern of structural and functional deterioration in USH2A-related RP [22].

Similarly, in our study, a positive correlation was observed between EZ width and BCVA, and a negative correlation was found between EZ width and disease duration in patients carrying *USH2A* variants. The predominance of USH2A variants in our cohort suggests that this gene may have an important contribution to RP pathogenesis and highlights its potential relevance for future gene-specific therapeutic strategies [23, 24].

In our cohort, age and BCVA were similar across the groups. SfCT was lower in patients with multiple variants compared to patients with variants detected in genes other than *USH2A* (*USH2A*: 215.5 μm; non-*USH2A* single-gene variants: 251.0 μm; multiple variants: 210.0 μm). These findings require further investigation in larger cohorts to better understand their clinical and biological significance. Additionally, *EYS* variants were identified in four patients, including three patients with variants in a single gene and one patient with variants in multiple genes.

Disease-causing variants of 16.3 kb in length containing the *PRPF31* gene (OMIM ∗606419, pre-mRNA processing factor 31), located on chromosome 19q13.4 and associated with autosomal dominant RP (adRP), represent the second most common cause of RP (RP11, OMIM #600138) [25]. Spagnuolo et al. reported that among 10 patients with RP1-associated RP, visual acuity was highly variable, the median age was 42 years, and the most common variant was c.2029 C > T (p.Arg677*). They identified cystoid macular edema (CME) in 25% and ERM in 10% of cases [26]. In our cohort, PRPF31 was detected in a single patient, who belonged to the multi-gene variants group and carried the c.403G > A (p.Ala135Thr, rs755833719) variant.

Bodenbender et al. conducted a study on a cohort of 85 patients diagnosed with retinitis pigmentosa (RP), who were found to be heterozygous for disease-causing variants in the *PRPF31* gene. The study reported that 20 patients (23.5%) exhibited symptoms consistent with CME [27]. In the present study, *PRPF31* was identified as the third most prevalent causative gene, with three patients exhibiting variants (two missense and one splice site). CME was observed in a single patient (33.3%).

Variants in the *RHO* gene have been reported to account for 30–40% of adRP cases, with Pro23His being particularly prevalent; these variants may cause protein folding or transport defects [18]. The most prevalent variant in Japanese, Italian, and Spanish cohorts was c.1040 C > T (p.Pro347Leu) [28–30], while studies from Belgium and the Netherlands reported p.Glu181Lys as the most common variant [31]. In the United States, p.Pro23His is most frequently observed [32]. In our cohort, *RHO* variants were identified in two patients (4.35%), both of whom also carried variants in other genes. The *RHO* variants were c.659T > G (p.Phe220Cys, rs766161322) and c.434 A > T (p.Asn145Ile), respectively.

*PDE6A* and *PDE6B* variants account for approximately 8% of arRP and induce photoreceptor apoptosis by disrupting cGMP metabolism [33–35]. In a study examining the clinical features and genetics of 16 RP patients with *PDE6A* variants, Hashem et al. reported a mean ASO of 11.6 years, with CMT ranging from 153 to 789 μm [36]. The authors attributed the early onset to the frequent presentation of the disease in childhood and the higher CMT values to the presence of CME in 56.3% of patients, reflecting a slow decrease in EZ width due to the gradually progressive nature of the disease [36].

In our study, *PDE6A* variants were identified in two patients with variants in a single gene, while a *PDE6B* variant was identified in one patient with variants in multiple genes. The mean ASO for these patients and other single-gene variant carriers was 32.2 years. The delayed onset that was observed in the present cohort may be ascribed to patient heterogeneity and divergent phenotypic expression. Furthermore, different variants within the same gene, or the same variant in different individuals, can result in variable phenotypes, potentially influenced by environmental factors (e.g., light exposure) and genetic modifier genes [19]. The CMT range in this group was 100–275 μm, which may be indicative of the limited number of patients with CME. It is noteworthy that one variant reported by Hashem et al., p.Arg102Ser, was also observed in our two PDE6A single-gene variant patients.

Artunay et al. reported on a patient with both Stargardt disease and RP who carried a single ABCA4 variant, thereby highlighting the impact of genetic variation on phenotypic expression [37]. In the present cohort, ABCA4 variants were identified in three patients. Notably, all three patients carried multiple gene variants.

CRB1 gene variants have been linked to a variety of ophthalmic phenotypes, including Leber congenital amaurosis, early-onset rod-cone dystrophy, RP, and cone-rod dystrophy. The proportion of arRP patients carrying CRB1 variants ranges from 0 to 6.5%, with an average of 2.7% [38, 39]. Consistent with these reports, *CRB1* variants were observed in two RP patients in our study (4.4%), one with variants identified in a single gene and the other with variants in multiple genes.

Iovino et al. presented a comprehensive review examining retinal and choroidal vascular changes in hereditary retinal diseases using OCTA [40]. They reported a marked decrease in SCP and DCP VD and alterations in FAZa (mostly increases, occasionally decreases), particularly in RP patients. These vascular changes were associated with functional parameters such as visual field loss, retinal sensitivity measured by microperimetry, and multifocal ERG (mfERG). For instance, decreases in DCP and CC VD were correlated with impaired macular function, while reductions in optic nerve head VD were associated with mean visual field deviation. The study highlighted genetic heterogeneity in vascular and structural parameters with RP-associated genes (e.g., *USH2A* or other RP-associated genes) showing variability in VD and retinal structural findings. However, this review did not provide specific genetic analyses of new patient cohorts, instead summarizing genetically heterogeneous cases from the existing literature. The authors concluded that OCTA is a valuable, non-invasive tool for detecting these changes and monitoring disease progression [40].

Changes in the CC and choroidal vasculature have been implicated in the pathogenesis of RP. Histopathological studies have demonstrated CC loss in vivo, and OCTA-based studies have revealed decreased choroidal blood flow in RP eyes [40–43]. Gesualdo et al. [44] reported a lower percentage of perfused choroidal area (PPCA) in 13 male patients (22 eyes) with RPGR-related X-linked RP compared to 10 healthy, age-matched controls. However, no significant correlations were identified between PPCA parameters and age, disease duration, BCVA, fundus autofluorescence, microperimetry sensitivity, or genotype in the patient group. In accordance with the hypothesis that CC hypoperfusion is the result of decreased metabolic demand due to primary photoreceptor loss, the inferior quadrant CC VD was more affected in patients with multiple gene variants in our study. With the exception of FAZa and inferior quadrant CC VD, all other VD parameters exhibited similar trends across the three groups. Furthermore, the groups did not differ significantly in terms of ASO or disease duration. Conversely, no disparities were identified in BCVA, OCT, or OCTA parameters among patients carrying missense versus frameshift variants. These findings suggest that variant type may not significantly influence the clinical presentation of RP, particularly with regard to retinal structural and vascular changes, or may not always determine the phenotypic expression of the disease. Furthermore, the presence of gene heterogeneity and differing disease stages among patients may have contributed to these results.

The present study has several strengths and limitations. A major strength of the study is the integration of detailed genetic analysis with multimodal retinal imaging, including OCT and OCTA, in patients with RP. The classification of patients according to genetically identified variants enabled genotype-oriented comparisons of structural and microvascular retinal changes among the study groups. In addition, the simultaneous evaluation of EZ integrity, retinal thickness parameters, and VD measurements provided a comprehensive assessment of retinal involvement in RP. The use of standardized imaging protocols and quantitative analyses further increased the reliability of the imaging findings. However, several limitations should also be considered. First, the retrospective design may limit the generalizability of the findings. Second, although 41 genetically characterized RP patients were included, OCT and OCTA subgroup analyses were performed in a relatively limited number of patients because only high-quality imaging data suitable for quantitative evaluation were analyzed. In addition, the marked genetic heterogeneity among RP-associated variants may have influenced subgroup comparisons. In addition, the interpretation of the multiple-gene variant group should be approached with caution, as the presence of variants in more than one RP-associated gene does not necessarily indicate additive or synergistic pathogenic effects without functional validation. Therefore, the findings related to this subgroup should be considered exploratory. Furthermore, ffERG data were not available for all patients due to the retrospective nature of the study. Another limitation is the inability to assess peripheral retinal and choroidal alterations because OCTA evaluation was performed using a 3 × 3 mm vascular density map. Finally, the cross-sectional design precluded longitudinal evaluation of structural and microvascular progression over time.

In conclusion, *USH2A* was the most frequently identified gene in patients with RP. When patients were grouped according to their genetic variants, preserved EZ, SfCT, and inferior quadrant CC VD were lower in patients with multiple gene variants compared to those with single-gene variants. Reduced retinal and choriocapillaris parameters, as well as choroidal circulation measures, were observed in patients harboring more than one variant. Overall, the genetic burden might appear to influence EZ integrity, CC metrics, and choroidal phenotypes. Taken together, these results emphasize the importance of comprehensive genetic analyses in RP patients for elucidating gene-specific phenotypic characteristics and potential treatment responses. To further elucidate the clinical implications of diverse genetic profiles, there is a compelling need for large-scale, multicenter studies with prolonged follow-up periods.

## Data Availability

The datasets generated and analyzed during the current study are available from the corresponding author on reasonable request. Patient data are de-identified to protect confidentiality and comply with institutional and ethical regulations.
